# Investigating ego modules and pathways in osteosarcoma by integrating the EgoNet algorithm and pathway analysis

**DOI:** 10.1590/1414-431X20165793

**Published:** 2017-02-16

**Authors:** X.Y. Chen, Y.H. Chen, L.J. Zhang, Y. Wang, Z.C. Tong

**Affiliations:** 1 Department of Orthopedics, The Affiliated Hospital of Xuzhou Medical College, Xuzhou, Jiangsu Province, China; 2 Department of Orthopedics, Affiliated Hospital of Shandong University of Traditional Chinese Medicine, Jinan, Shandong Province, China; 3 Department of Orthopedics, The 5th People's Hospital of Jinan, Jinan, Shandong Province, China; 4 Department of Orthopedics, The Third Affiliated Hospital of the Second Military Medical University, Shanghai, China; 5 Department of Bone Oncology, Xi'an Honghui Hospital, Xi'an, Shaanxi Province, China

**Keywords:** Osteosarcoma, Ego, Genes, Modules, Pathways

## Abstract

Osteosarcoma (OS) is the most common primary bone malignancy, but current therapies are far from effective for all patients. A better understanding of the pathological mechanism of OS may help to achieve new treatments for this tumor. Hence, the objective of this study was to investigate ego modules and pathways in OS utilizing EgoNet algorithm and pathway-related analysis, and reveal pathological mechanisms underlying OS. The EgoNet algorithm comprises four steps: constructing background protein-protein interaction (PPI) network (PPIN) based on gene expression data and PPI data; extracting differential expression network (DEN) from the background PPIN; identifying ego genes according to topological features of genes in reweighted DEN; and collecting ego modules using module search by ego gene expansion. Consequently, we obtained 5 ego modules (Modules 2, 3, 4, 5, and 6) in total. After applying the permutation test, all presented statistical significance between OS and normal controls. Finally, pathway enrichment analysis combined with Reactome pathway database was performed to investigate pathways, and Fisher's exact test was conducted to capture ego pathways for OS. The ego pathway for Module 2 was CLEC7A/inflammasome pathway, while for Module 3 a tetrasaccharide linker sequence was required for glycosaminoglycan (GAG) synthesis, and for Module 6 was the Rho GTPase cycle. Interestingly, genes in Modules 4 and 5 were enriched in the same pathway, the 2-LTR circle formation. In conclusion, the ego modules and pathways might be potential biomarkers for OS therapeutic index, and give great insight of the molecular mechanism underlying this tumor.

## Introduction

Osteosarcoma (OS), the most common primary bone malignancy, derives from primitive bone-forming mesenchymal cells (1), and has an annual worldwide incidence of approximately 1-3 cases per million (2), occurring most commonly in the metaphyseal regions of long bones in adolescents and young adults, but also in patients over 40 years of age (3). The standard curative osteosarcoma treatment is surgery, but survival is approximately 15–17% (4). Even though the survival rate has improved considerably after the introduction of neoadjuvant chemotherapy, the need for advances in treatment regimens is still high (5). A better knowledge on biological markers and pathology of OS may help provide new treatments for this tumor (6).

High-throughput experimental technologies have been applied to explore diagnostic gene signatures and biological processes of human diseases (7). This technology may provide novel insights to the underlying pathological mechanisms of OS. Genes in certain diseases do not work alone, often co-operating with each other, and together participating in functional biology. Thus, one could evaluate significant genes and biological processes and their association with disease using a network strategy, especially protein-protein interaction (PPI) networks (8). Besides, networks also can provide significant instructions for uncovering unknown connections in incomplete networks. Although the data of large-scale protein interactions is accumulating with the development of high throughput testing technology, a certain number of significant interactions have not been tested, such as key genes in certain pathways (9). This type of difficulty might be resolved to some extent by utilizing sub-networks or modules of the complex network (10). Ning et al. (11) identified pathway-related modules in high-grade OS based on topological centralities analyses of co-expression networks and sub-networks, and made contributions in understanding the molecular pathogenesis of high-grade OS and identifying potential biomarkers for effective therapies. However, studies focusing on OS are rare and not sufficient to support the urgent needs.

Therefore, we aimed to identify ego modules and pathways in OS by integrating EgoNet algorithm and pathway enrichment analysis. The EgoNet algorithm identifies significant sub-networks called ego modules that are functionally associated with diseases, as well as accurately predict clinical outcomes (12,13). An ego module is the part of the network that involves a specific node called ego, and consists of a neighborhood including all nodes to which the ego is connected at a certain path length. The EgoNet algorithm has been used for investigating module over-representation analysis in ConsensusPathDB (14), which validates the feasibility of this method. Ego modules are functionally associated with diseases, and accurately predict clinical outcomes (12), consisting of a systemic way to study the pathological mechanism underlying OS at molecular level. Moreover, pathway analysis has become the main method for gaining insight into the underlying biology of genes and proteins, as it reduces complexity and has increased explanatory power (15).

## Material and Methods

**Figure 1:**
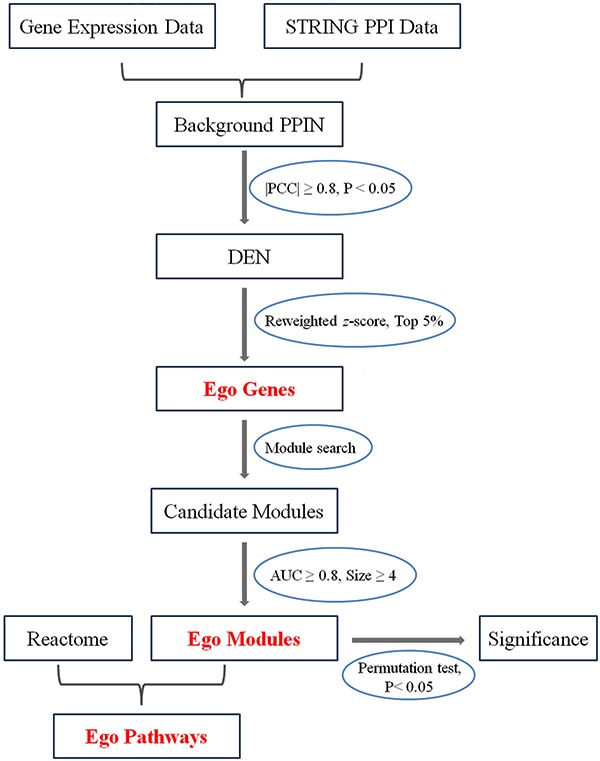
Flow chart for identification of ego genes, modules and pathways in osteosarcoma. PPI: protein-protein interaction; PPIN: PPI network; DEN: differential expression network; AUC; area under the receiver operating characteristic (ROC) curve.

To identify ego modules and pathways in OS, we integrated the EgoNet algorithm and pathway-related analysis, as shown in Figure 1. The EgoNet algorithm identifies ego modules from gene expression and large-scale biological networks (12). It comprises four steps: constructing the background PPI network (PPIN) based on gene expression data and PPI data; extracting the differential expression network (DEN) from the background PPIN; identifying the ego genes according to topological features of genes in reweighted DEN; and collecting the ego modules using module search by ego gene expansion. Subsequently, the permutation test was implemented to evaluate the statistical significance of ego modules. Finally, pathway enrichment analysis based on the Reactome database and the Fisher's exact test was conducted to investigate ego pathways for OS.

### Gene expression and PPI data recruitment

Microarray gene expression profile with accessing number E-GEOD-36001 (5) was recruited from the online ArrayExpress database (http://www.ebi.ac.uk/arrayexpress/). E-GEOD-36001 was comprised of 19 OS samples and 6 normal samples, and was deposited on A-MEXP-930 - Illumina Human-6 v2 Expression BeadChip Platform (Illumina, USA). In order to control the quality of this data, standard pre-treatments were conducted, including background correction (16), normalization (17), probe correction (18) and summarization (16). As a consequence, 19,032 genes were selected from the gene expression data for further analysis.

Mapping disease-associated genes to interacted data can greatly empower the understanding of disease mechanisms in contrast to studying individual genes (19). Therefore, we integrated the gene expression data into a confirmed PPIN and gained a more reliable PPIN denoted as background PPIN. The confirmed PPIN with 16,730 genes and 787,896 interactions was acquired from the Search Tool for the Retrieval of Interacting Genes/Proteins (STRING) database (http://string-db.org/) (20). There were 8,238 genes and 51,258 interactions in the background PPIN for exploitation in a subsequent study.

### Ego genes selection

With the purpose of removing indirect and indistinctive interactions in the background PPIN, Person correlation coefficient (PCC) was implemented to assess the edge scores, which evaluates the probability of two co-expressed gene pairs (21). One side *t*-test (22) was employed to calculate the P values for the scores between OS samples and normal controls. Only those which met the thresholds of |PCC| ≥ 0.8 and P<0.05 were retained, termed DEN.

Subsequently, we calculated the topological feature for every gene in the DEN using the function *f* (23): 
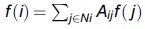
 where *N*(*i*) represents the set of neighbors of gene *i*; A_*ij*_ is the degree of normalized weighted adjacency matrix, computed as 
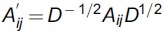
 ; where *D* is a diagonal matrix with element 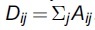 . Hence the significance of a node depends on the number and importance of its neighbors, and the strength of the connection (24). Based on the *f*(*i*), a *z*-score for each gene was computed (25). All nodes in DEN were ranked in descending order of their *z*-scores, and the top 5% were selected as ego genes.

### Ego modules identification

In this step, module search by ego gene expansion was conducted to extract modules from the DEN, which iteratively involved genes whose addition led to the maximum increase in the prediction accuracy of the model until the prediction accuracy dropped (12). Meanwhile, the prediction accuracy capability of a module was evaluated by the area under the receiver operating characteristics (AUC) curve implemented in support vector machines (SVM) model (26). The AUC has been denoted as a better measure for assessing the predictive ability of machine learners than the assessment by clinical classification performance (27).

Taking each ego gene (*v*) as a module *M* = {*v*}, for each vertex *u* in its neighborhood, the new module M = {*v*}, and the AUC entropy increase between *M* and *M′* was defined as follows: 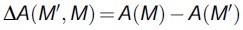 Δ*A*(*M′*,*M*) >0 indicated that the addition of vertex *u* improved the AUC of the former module *M*. This expansion process spread outward from the ego node progressively to involve more genes in the DEN and stopped when the AUC of the candidate module dropped. Candidate modules with AUC ≥0.8 and gene size ≥4 were considered to be ego modules.

### Statistical analyses

The permutation test was utilized to evaluate the statistical significance of ego modules between OS patients and normal controls, which examines the significance of effects in un-replicated factorial experiments and its stated test size without any distributional requirements (28,29). The permutation test was performed 1000 times for each ego module, and their AUC values were also obtained. Meanwhile, we evaluated the possibility of the AUC for the ego module identified by EgoNet algorithm being smaller than that found by the permutation test, as the P value for the ego module. Multiple testing in the Benjamini-Hochberg method was employed to adjust these P values (30). Only ego modules with P<0.05 were considered to have a significant difference between OS samples and normal samples.

### Ego pathways exploration

Generally, interacting genes tend to work together and participate in similar biological activities, and hence we explored pathways enriched by ego module genes based on the Genelibs (http://www.genelibs.com/gb/index.jsp) for pathway enrichment analysis. First, a confirmed pathway database, Reactome (http://www.reactome.org/), was selected to capture all biological pathways for human beings. A total of 1675 pathways were obtained. Subsequently, to make these pathways more correlated to OS, we combined the intersections with background PPI data. Pathways of intersected genes ranging from 5 to 100 were retained as our study objectives, termed background pathways. There were 1136 background pathways for OS.

By mapping ego module genes to the background pathways, the corresponding pathways were obtained and then their enrichment effects were evaluated using the Fisher's exact test (*F*) (31). For gene *i*, *F* ^(*i*)^ was computed:



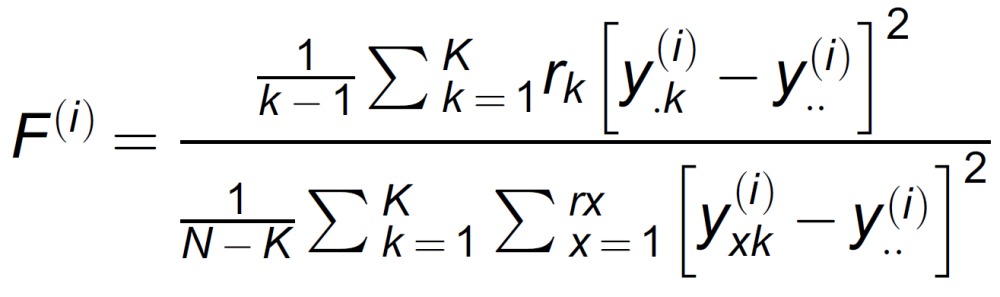
(1)where *x* represents the corresponding expression value in each replicate sample; *r_k_* the corresponding expression for each cell type *k* = 1, …, *K*; *y* is the mixed effect model; and *N* is the total number of samples. During this process, P values for each pathway were calculated, and then adjusted by the Benjamini-Hochberg method (30). Only a pathway with P<0.05 was regarded as an ego pathway for the ego module.

## Results

### Ego genes

**Figure 2:**
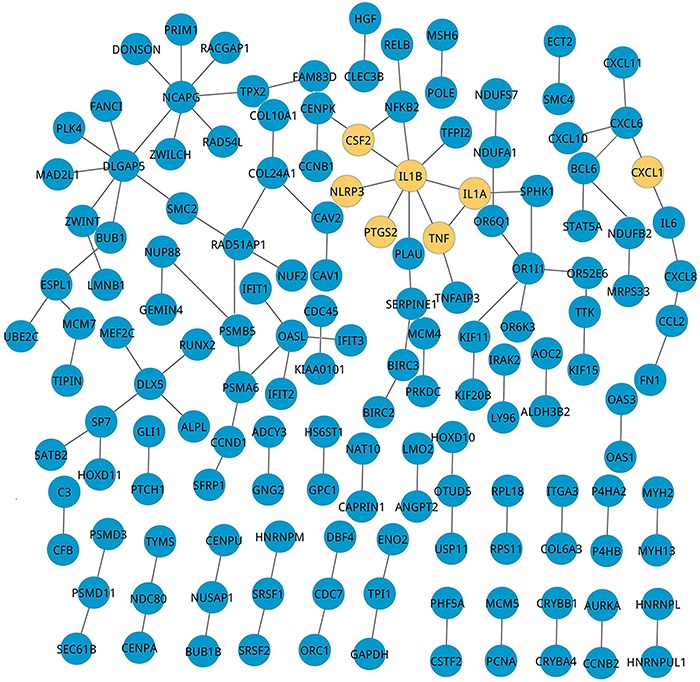
Differential expression network (DEN) for osteosarcoma. Nodes represent genes, and edges are interactions between any two genes. The yellow ones were selected as ego genes.

In the current study, we constructed a background PPIN with 8,238 genes and 51,258 interactions based on gene expression data and STRING PPI data, and extracted the DEN from background PPIN by setting |PCC| ≥0.8 and P<0.05. The DEN (Figure 2) had 149 nodes and 288 edges. Next, we reweighted genes in DEN according to their topological features, ranked them in descending order of their *z*-scores, and defined the top 5% as ego genes. A total of 7 ego genes were obtained, *IL1B* (*z*-score=2.41), *IL1A* (*z*-score=2.14), *NLRP3* (*z*-score=2.08), *TNF* (*z*-score=2.04), *PTGS2* (*z*-score=1.73), *CXCL1* (*z*-score=1.42), and *CSF2* (*z*-score=1.35) (Table 1).

**Table:**
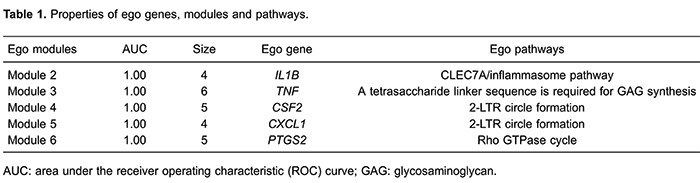

### Ego modules

**Figure 3:**
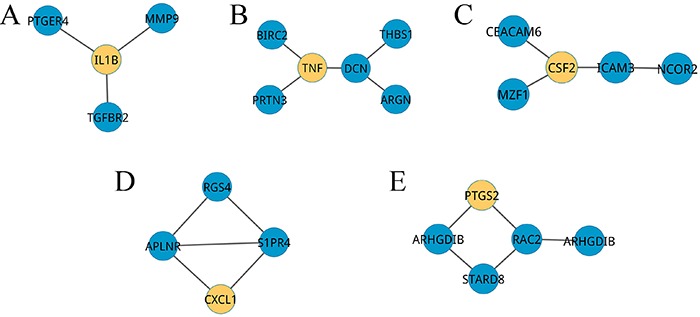
Ego modules. *A*, Module 2; *B*, Module 3; *C*, Module 4; *D*, Module 5; and *E*, Module 6. Nodes are genes, and edges represented interactions between any two genes. The yellow nodes are the ego genes of the modules.

Every ego gene had the corresponding candidate module, and thus 7 candidate modules were obtained. When setting the thresholds of AUC ≥0.8 and gene size ≥4, the candidate Module 1 (AUC=0.75, size=2) and Module 7 (AUC=0.94, size=3) were removed. The retained 5 candidate modules were denoted as ego modules, and their properties are displayed in Table 1. The 5 ego modules (Modules 2, 3, 4, 5 and 6) had the highest AUC of 1.00. However, their gene compositions were greatly different, as shown in Figure 3. Module 3 possessed the largest gene size, including *TNF* (ego gene), *DCN*, *PRTN3*, *BIRC2*, *ARGN* and *THBS1*, and the 5 genes made up 5 interactions.

The permutation test was carried out 1000 times for each ego module. The results showed that all of the 5 ego modules had statistical significance, which suggests that these ego modules play key roles in the progression of OS.

### Ego pathways

The ego pathway for Module 2 was CLEC7A/inflammasome pathway (P=4.69E-02), while for Module 3 was A tetrasaccharide linker sequence, required for GAG synthesis (P=4.97E-02), and for Module 6 was Rho GTPase cycle (P=2.78E-02). Interestingly, genes in Modules 4 and 5 were enriched in the same pathway, the 2-LTR circle formation (P=0.04).

## Discussion

The EgoNet is a general framework for ego module selection, and can be readily applied to datasets with continuous, multi-class, and survival outcome variables. The key advantage of EgoNet algorithm is its capability to discover potential markers that are not differentially expressed, but are functionally associated with many differentially expressed genes (12), providing a systematic way to study the pathological mechanism underlying OS at molecular level. Therefore, in the present work, we applied EgoNet to explore ego modules in OS, and further identified ego pathways for these ego modules.

A total of 5 ego modules (Modules 2, 3, 4, 5 and 6) with AUC=1.00 were obtained, indicating a good classifying performance between OS and normal groups. The results of the permutation test showed that all Modules were significant between OS and normal state, which suggests they are more important in the progression of OS.

The ego gene for Module 2 was *IL1B* (Interleukin 1 beta), an important mediator of the inflammatory response, involved in a variety of cellular activities, including cell proliferation, differentiation, and apoptosis (32). In addition, inflammation is associated with cancer risk and development; there is evidence that a pro-inflammatory environment promotes activation of *IL1B* (33). We uncovered that the ego pathway for the Module 2 was CLEC7A/inflammasome pathway, which enables the host immune system to mount a protective T-helper 17 cells (TH17) response against infection. The inactive precursor pro-IL1B has to be processed into mature bioactive form of *IL1B* and is usually mediated by inflammatory cysteine protease caspase-1. Gringhuis et al. showed that C-type lectin domain family 7 member A (*CLEC7A*)-mediated processing of *IL1B* occurs possibly through its triggering inducing a primary noncanonical caspase-8 inflammasome for pro-*IL1B* processing (34). Moreover, multiple studies demonstrated that *IL1B* is related to many human cancers (35). Hence, we might infer that the Module 2 and its ego pathway are closely correlated to OS.

It has been reported that the addition of TNF-α (tumor necrosis factor α) and *IL1B* simulated inflammation in OS cell line (36). In our study, *TNF* was the ego gene for Module 3, which validated the feasibility and confidence of our results to some extent. TNF is a multifunctional pro-inflammatory cytokine involved in the regulation of a wide spectrum of biological processes including cell proliferation, differentiation, apoptosis, lipid metabolism, and coagulation, and has been implicated in a variety of diseases, such as autoimmune diseases, insulin resistance, and cancer (37). It had been reported that *TNF-α* indirectly increased bone sialoprotein expression in human osteosarcoma cell line Saos2 (38). Further, *TNF* is associated with increased risk of OS. Liu et al. (39) suggested that ampelopsin inhibited the TNF-α-induced migration and invasion of OS cells. Interestingly, the ego pathway for Module 3 was A tetrasaccharide linker sequence required for GAG synthesis. In general, the biosynthesis of dermatan sulfate/chondroitin sulfate and heparin/heparan sulfate GAGs starts with the formation of a tetrasaccharide linker sequence to the core protein (40)

After this process, the next hexosamine addition is critical as it determines which GAG is formed, and the alteration of the progression perhaps leads to protein formation disorders, which could lead to cancer. Therefore, Module 3 and its enriched ego pathway had tight relationship with OS. These findings also suggest that each ego module does not act individually, and two or more may co-regulate certain functions in the process of OS. The inference was confirmed by the same ego pathway in Modules 4 and 5.

In conclusion, we have successfully identified 5 ego modules and 5 ego pathways for OS based on the EgoNet algorithm and pathway enrichment analysis. These findings might be potential biomarkers for OS therapeutic index, and provide insights into the molecular mechanism underlying this tumor. How these ego modules co-operated with each other, however, still remains unclear, and further specific investigations are indispensable. The current study was based only on bioinformatic methods, and lacked experimental verifications. Thus, efforts should be directed to converting these theoretical results into clinical practice in the future.
